# Engineering vascularized skeletal muscle tissue with transcriptional factor ETV2-induced autologous endothelial cells

**DOI:** 10.1007/s13238-018-0542-7

**Published:** 2018-04-23

**Authors:** Guanrong Yan, Ruibin Yan, Cheng Chen, Cheng Chen, Yanqiu Zhao, Wei Qin, Matthew B. Veldman, Song Li, Shuo Lin

**Affiliations:** 1 Laboratory of Chemical Genomics, School of Chemical Biology and Biotechnology, Peking University Shenzhen Graduate School, Shenzhen University Town, Shenzhen, 518055 China; 2 Department of Molecular, Cell and Developmental Biology, University of California Los Angeles, Los Angeles, CA 90095-1555 USA

**Dear Editor,**

Newly engineered tissues often fail to function due to insufficient blood vessels formation. Autologous vascular endothelial cells are ideal sources for tissue engineering but are often of limited availability. We first identified that transcriptional factor *Etv2* (ets variant 2, initially named as *Etsrp*) was specifically expressed in the zebrafish vascular endothelial cells and was required for vascular development in zebrafish (Sumanas and Lin, 2006). ETV2 has been found to increase endothelial differentiation of human and mouse embryonic stem cells as well as to directly transdifferentiate somatic cells into endothelial-like cells (Lindgren et al., 2015; Morita et al., 2015). In zebrafish embryos, overexpression of *Etv2*/*Etsrp* induced vascular gene expression and converted fast skeletal muscle cells into endothelial cells that were incorporated in functional blood vessels (Veldman et al., 2013). This finding suggests that ETV2 could be potentially used to generate autologous endothelial cells from human skeletal muscle cells for regenerative medicine. It is worth noting that protocols for skeletal muscle cells isolation and *ex vivo* expansion have been well established, allowing efficient production of muscle satellite cells/myoblasts from minimally tissue invasive (Webster et al., 1988). Therefore, selection of skeletal muscle cells as an alternative isogenic source for generating endothelial cells has practical advantages and benefits.

In this study we compared capacity of several human cell types to be induced to express endothelial genes by ETV2 and showed that human skeletal muscle cells (HSkMCs) are highly amendable for this endothelial induction. We first detected the potential of endothelial transdifferentiation induced by ETV2 overexpression in five human cell types. Cells used in this experiment were human skeletal muscle cells (HSkMCs), adipose-derived mesenchymal stem cells (ADMSCs), umbilical cord-derived mesenchymal stem cells (UCMSCs), human embryonic lung fibroblast cells (HFL-1) and human skin fibroblast cells (HSFs). All these cells were infected with equal numbers of ETV2 lentivirus. Following ETV2 lentivirus infection, cell morphologies and expression of endothelial specific genes were analyzed. The infected cells all appeared to change morphologies into an endothelial-like shape. Among them, ETV2-HSkMCs showed the most uniform conversion, similar to the morphology of human umbilical vein endothelial cells (HUVECs) (Fig. 1A). Again using HUVECs as a control, the expression of endothelial cell markers was measured. Flow cytometry (FCM) analysis revealed that more than 50% of ADMSCs, UCMSCs, HFL-1, HAFs and HSkMCs expressed endothelial gene marker CDH5/VE-cadherin/CD144. Surprisingly, under the same condition, over 90% of HSkMCs were converted to express CDH5. Similar to CDH5, VEGFR-2 positive population were significantly induced after ETV2 expression, with the highest efficiency in HSkMCs (Figs. 1B and S1).

**Figure 1:**
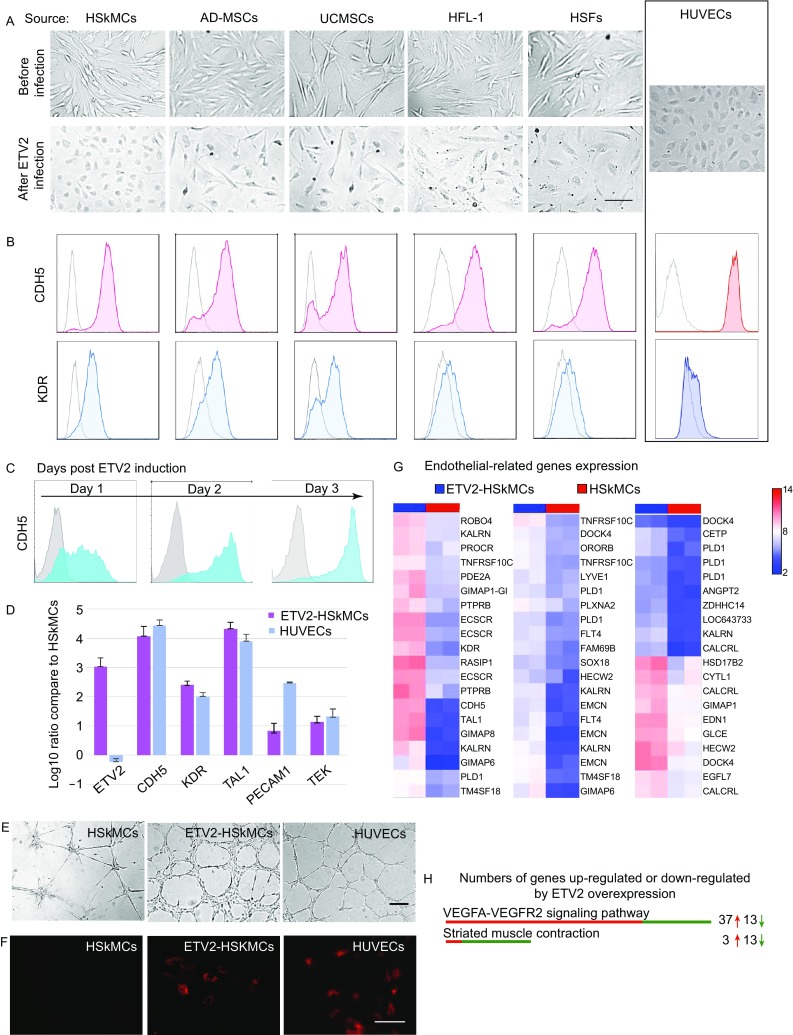
**Analysis of endothelial conversion after ETV2 transduction**. (A and B) Comparison of endothelial potential after ETV2 transduction. (A) Before conversion, these cells had diverse morphologies. 5 days after ETV2 reprogramming, cobblestone-like shape similar to HUVECs became apparent. Scale bar: 100 μm. (B) After ETV2 lentivirus infection, HSkMCs, ADMSCs, UCMSCs, HFL-1, HSFs expressed endothelial markers CDH5 and KDR. (C) Temporal expression profile of endothelial maker CDH5 after ETV2 infection. (D) Expression of endothelial related genes in HUVECs and HSkMCs induced by ETV2 at 3 dpi, compared to normal HSkMCs. Fold-change is expressed in a log10 scale. (E) Tube assay of ETV2-HSkMCs (3 dpi) compared to HUVECs. Scale bars: 200 μm. (F) After 3 dpi, uptake of a-LDL was detected in ETV2-HSkMCs and HUVECs, but not in HSkMCs. Scale bars: 50 μm. (G) Previously reported endothelial specific genes were selected for comparison and ETV2 expression in HSkMCs induced up-regulation of these genes. (H) Wikipathway analysis of microarray data from control and ETV2-HSkMCs revealed that 37 out of 50 (75%) of differentially expressed genes in the VEGF-VEGFR2 pathway were upregulated, while 13 out of 16 (81%) of differentially expressed genes in the striated muscle contraction pathway were downregulated

Based on the findings above and early evidences in zebrafish we hypothesized that the HSkMCs can be a good resource for endothelial transdifferentiation. First, the time course of CDH5 induction by ETV2 was investigated. Flow cytometry showed that over half of the cells already expressed CDH5 at 1 day post infection (dpi) and about 90% at 3 dpi (Fig. 1C). Under the same condition, ETV2-ADMSCs transdifferent more slowly and less efficient in responding to ETV2, only about 50% expressed CDH5 at 3 dpi (Fig. S2). The relatively fast response of HSkMCs to endothelial induction by ETV2 can be a benefit for the use of this cell type in regenerative medicine. We then expanded molecular analysis of endothelial transdifferentiation induced by ETV2. By qRT-PCR, we confirmed that overexpression of ETV2 induced expression of multiple vascular genes, including KDR, TAL1, PECAM1 and TEK (Fig. 1D). ETV2-HSkMCs showed partial positive of CD34, which is similar to HUVECs (Fig. S3). Next, we tested the ability of HSkMCs to form vascular tubes *in vitro* with or without ETV2 expression. The results showed that ETV2-HSkMCs formed vascular-like tubes with similar size and structure of HUVECs. Although HSkMCs alone also formed fiber-like structures, they appeared different from that of HUVECs (Fig. 1E). Tube formation assay demonstrates that ETV2 initiates morphological changes resembling endothelial process *in vitro*. As another functional test, we assayed the level of LDL uptake in these cells. As shown in Figure 1F, after 3 dpi, ETV2- HSkMCs had acquired the ability to take up DiI-acetylated low-density lipoprotein (DiI-Ac-LDL) similar to the endothelial cells but this was not detected in HSkMCs.

To evaluate the transcriptome-wide effects of ETV2 expression in HSkMCs, we performed microarray studies on HSkMCs and ETV2-HSkMCs. Through calculating the relative gene expression level, we identified 694 transcripts with over 4-fold changes and 3,897 transcripts with over 2-fold changes. First, when comparing HSkMCs, ETV2-HSkMCs and independent various endothelial cells microarray data (Aranguren et al., 2013), heatmap analyses showed that ETV2-HSkMCs were closer to endothelial cells than HSkMCs (Fig. S4). Second, ETV2-HSkMCs showed robust upregulation of vascular related genes and concomitant downregulation of muscle related genes than HSkMCs. Specifically, we selected some endothelial specific genes based on bioinformatics analysis by Bhasin et al. (2010) and those up-regulated genes after ETV2 expression is shown as a heatmap in Fig. 1G. And skeletal muscle functional genes enriched in several downregulated clusters are shown in Fig. S5B and S5C. Third, Wikipathway analysis showed universal changes in VEGF-VEGFR2 and striated muscle contraction pathway (Figs. 1H and S5A). PI3K/AKT pathway, another endothelial cell differentiation related pathway, was also highly induced in ETV2-HSkMCs (Fig. S5D). Overall, transcriptome analysis suggests that ETV2 induced trans-differentiation of endothelial cells likely involves activating the VEGF-VEGFR2 and PI3K/AKT pathways.

This direct trans-differentiated process is different from induced pluripotent stem cell generation in that there is no pluripotency stage. The major advantages of the induced endothelial-like cells described here are simple, fast and efficient. These highly enriched endothelial-like cells could be directly used in cell based therapies or tissue engineering, reducing the risk of contamination from additional cell purification procedures. Since these endothelial-like cells are induced in a relatively short window of time, they most likely represent endothelial cells at early stage. Overall, these studies suggest that ETV2-HSkMCs are readily converted to ETV2-induced endothelial-like cells (ETV2-iEC).

Skeletal muscle tissue is highly vascularized and accounts for approximately 40% of the body mass in human (Frontera and Ochala, 2015). Muscle defects caused by traumatic events, diabetic tissue damage or surgical operation such as tumor removal could result in muscle dysfunction and physical deformities. The current choices of treatments are limited and inefficient. One promising therapeutic option is to implant pre-vascularized engineering muscle tissue (Koffler et al., 2011; Levenberg et al., 2005). In most cases, self-vascularization by host or supplement of endothelial cells directly harvested from host is not adequate for tissues of large volumes. Without blood vessel formation, the newly generated tissues will fail to function due to insufficiency of oxygen and nutrition supply (Griffith and Naughton, 2002; Novosel et al., 2011). Thus, artificial reprogramming of autologous somatic cells into vascular endothelial cells gives considerable new hope to overcome this longstanding obstacle.

Thus, we thought muscle tissue could be used as a complex model to test the ability of our ETV2-iEC in forming vessels. To determine if ETV2-induced endothelial-like cells (ETV2-iEC) could contribute to vascularization of engineered muscle tissue for transplantation, we adopted the procedures shown in Figs. 2A and S6A. Both synthetic matrix and biological materials were used. Decellularized scaffolds are produced by removing cellular components and antigenicity from natural tissue but the procedure lacks standardization and reproducibility (Badylak et al., 2011; Novosel et al., 2011). The biological scaffolds we used were from *Cavia porcellus* (Guinea pig) skeletal muscle tissue and generated through detergent perfusion and enzymatic digestion, which removed all cellular material while maintained the three-dimensional networks of collagen and elastic fibers (Fig. S6B and S6C). Synthetic materials like PLLA (poly-(L-lactic acid)) and PLGA (polylactic-glycolic acid) provide physical support for cells with highly porous biodegradable structures that are easy to operate and suitable for large-scale production (Levenberg et al., 2002; Levenberg et al., 2003). The synthetic scaffolds we used were composed of 50% PLLA and 50% PLGA with an average pore diameter of 250 μm, which were fabricated through a salt-leaching process (Fig. S7). Thus we engineered muscle tissue using HSkMCs with/without ETV2-induced endothelial-like cells on the synthetic polymer scaffolds or the naturally decellularized muscle scaffolds through the periods of *in vitro* culture and *in vivo* implantation.

**Figure 2:**
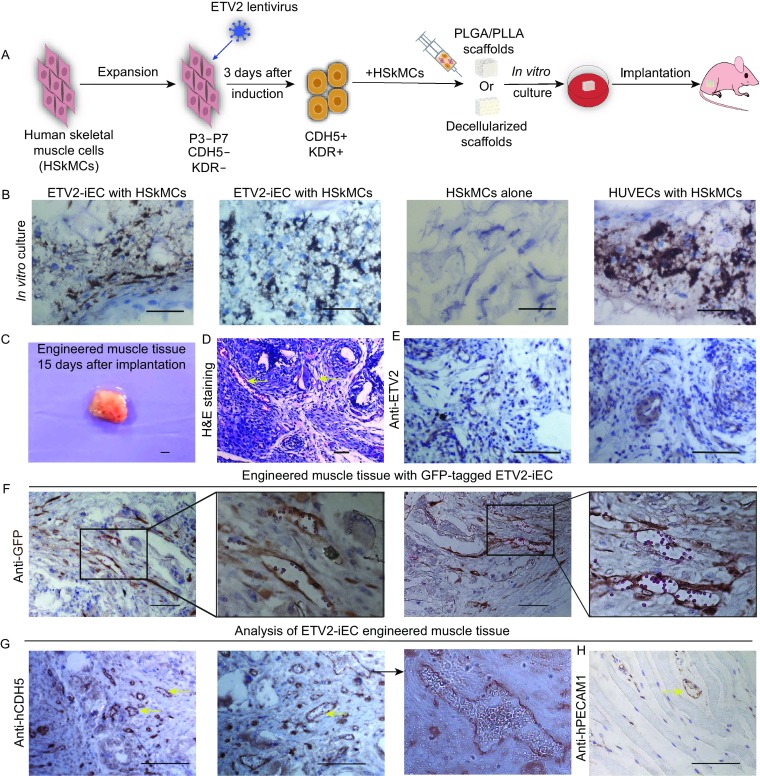
**Analysis of engineered muscle tissue with ETV2-iEC on PLLA/PLGA scaffolds**. (A) Experimental design for muscle tissue engineering with ETV2-iEC. (B) Analysis of engineered muscle tissue with ETV2-iEC on PLLA/PLGA scaffolds *in vitro*. DAB staining of CDH5 showed vessel-like structure formation after *in vitro* culture with ETV2-iEC or with HUVECs, while HSkMCs alone had no staining signal. (C–H) *In vivo* analysis of engineered muscle on PLLA/PLGA scaffolds. (C) Engineered tissue dissected out from nude mice 15 days after implantation. (D) H&E staining. (E) Immunostaining against ETV2 traced ETV2 expression. (F) Rabbit GFP mAb showed that some GFP-ETV2 infected cells were placed around vessels and harbored oval biconcave blood cells in engineered muscle. By brief eosin staining for one second, red blood cells could be stained as red, distinct from the brown signal of DAB. Immunostaining against human CDH5 (G) and human PECAM1 (H). Yellow arrows indicate vessel structures and black arrow indicates blood cells in magnified views. Scale bars: 100 μm

First, the ability of these scaffolds to support tissue growth *in vitro* was tested. Approximately 1.5 × 10^6^ cells per scaffold, half were CDH5 positive cells and the rest were skeletal muscle cells, were injected into the scaffolds and allowed to grow under tissue culture condition. After 5 days of culture in decellularized scaffolds or 7 days of culture in PLLA/PLGA scaffolds, the status of engineered muscle tissue was analyzed by sectioning and histological staining. The results showed that CDH5 positive cells that organized into vessel-like structures were only detectable in the muscle tissue mixed with ETV2-iEC or HUVECs but not in the cell mass by HSkMCs alone (Figs. 2B and S6D). This finding suggests that, similar to HUVECs, ETV2-iEC contributed to vascular formation when mixed with skeletal muscle cells. More importantly, if only HSkMCs were seeded in these scaffolds, the cells did not differentiate into CDH5 positive cells and form any vessel like structures.

Next, we analyzed vascularization of muscle tissue engineered with ETV2-iEC *in vivo*. Blocks of hind leg muscle of nude mice were surgically removed and replaced with our engineered tissue in the same position. Fifteen days after engineered muscle tissue implantation, they were removed and analyzed histologically (Fig. 2C). H&E staining revealed that tissue generated by co-injection of HSkMCs and ETV2-iEC survived in PLLA/PLGA and had formed many vessels (Fig. 2D). Immunohistochemistry results revealed positive ETV2 expression abundantly around the vessel, supporting an inductive role of ETV2 in forming new vessels (Fig. 2E). ETV2-GFP lentivirus were used to produce GFP-tagged ETV2-induced endothelial cells for engineered muscle tissue. This confirmed that our ETV2-iEC had contributed to functional vessels formation which enclosed circulating red blood cells (Fig. 2F). Immunohistochemistry staining using human specific anti-CDH5 antibody indicated that these human CDH5 positive cells were further incorporated into functional vessels in nude mice with red blood cells inside, indicating successful blood circulation (Fig. 2G). Similar results were obtained from tissue grown in decellularized scaffolds (Fig. S6E and S6F). It seems that the types of scaffold materials did not significantly affect angiogenesis induced by ETV2-iEC. Anti-human nuclei antibody staining confirmed that human cells contributed to the blood vessels (Fig. S8). Muscle tissue with ETV2-iEC also contained some human PECAM1 positive cells in the vessel area, indicate the maturation of these endothelial-like cells (Figs. 2H and S6G). Thus, we proved that ETV2-iEC contributed to functional blood vessels formation in engineered muscle. This finding suggests possibility of engineering isogenic vascularized muscle tissue using a single source of patient-specific skeletal muscle cells.

In conclusion, our studies indicate that ETV2-HSkMCs contribute to vessel formation *in vitro* and *in vivo*, and may have potential to be developed as an autologous endothelial cell source for clinical applications.

## Electronic supplementary material

Below is the link to the electronic supplementary material.

Supplementary material 1 (PDF 1065 kb)
