# Supplementary material for: Engineering vascularized skeletal muscle tissue with transcriptional factor ETV2-induced autologous endothelial cells
Source: Protein Cell. 2018 Apr 23;10(3):217–22. doi: 10.1007/s13238-018-0542-7 (PMC6338622; doi:10.1007/s13238-018-0542-7)
Supplement: Supplementary file 1 — Supplementary material 1 (PDF 1065 kb) [file 13238_2018_542_MOESM1_ESM.pdf]

Supplemental material and methods

Cell Culture

Several commonly used cell types were used in this experiment to compare with Human skeletal muscle cells (HSkMCs) were purchased from ScienCell Research Laboratories (Cat.3500), which were isolated from human trapezius muscle and erector spinae muscles of the back, and cultured in Skeletal Muscle Cell Medium (SkMCM, Cat.3501). Adipose derived mesenchymal stem cells (ADMSCs) and umbilical cord-derived mesenchymal stem cells (UCMSCs) were expanded in Mesenchymal Stem Cell Medium (ScienCell, Cat.7501). Human embryonic lung fibroblast line HFL-1 cells (BNCC337730) and human skin fibroblast cells (BNCC338008) were grown in Dulbecco's Modified Eagle Medium (HyClone) with 15 % FBS. Human umbilical vein endothelial cells (HUVECs) and ETV2 infected cells were maintained in Endothelial Cell Basal Medium (EBM-2, Lonza) supplemented with EGM-2 bullet kit. Cells were cultured at 37°C in 5 % CO2. Medium was changed every 2 days and cells were detached using Accutase (Sigma).

Lentivirus production and transduction

Lentiviral vectors containing cDNA of ETV2 under the control of EF1a promoter (Lindgren et al., 2015) with or without GFP tag were co-transfected with the 2rd generation packaging systems (addgene) in 293T cells. Viral supernatants were harvested 48 h and 72 h after transfection and ultra-centrifuged to concentrate the virus. In the reprogramming process, cells were seeded in 12-well plate at $2.5*10^4$ cells per well and cultured in their recommended condition. 24 h later, cells were infected with equal amount of ETV2-lentivirus, supplemented with 5 ug/ml polybrene. 4 hours later, lentivirus were removed and washed by PBS. Cells were continued to grow in EGM-2 endothelial cell medium (Lonza) with the same culture condition.

Flow cytometry analysis

Flow cytometry was performed using FACS Calibur (BD Biosciences). The following antibodies from BD Biosciences were used: Anti-human CD144/ VE-cadherin/CDH5 (Cat.561567), Anti-human CD309/VEGFR-2/KDR (Cat.560494), Anti-human CD34 (Cat.555821) and isotype control Cat.555748 & Cat.557714.

qRT-PCR

RNA expression of specific genes was quantified using quantitative RT-PCR (qPCR). Total RNA was isolated by RNA isolation kit (Life Technologies Cat.AM1914). After removing genomic DNA, reverse transcription was performed using Takara kit (Cat.RR047A). Real-time PCR was performed using SYBR Green (Roche) on Bio-Rad CFX96. Relative gene expression levels of ETV2-HSkMCs or HUVECs were normalized by comparison to GAPDH and ACTB as an internal control, and

were calculated relative to polybrene treated HSkMCs groups. The log10 rati transformed data is shown. Gene-specific primers are listed in Supplementary table 1.

Vascular tube formation

10^4 cells were resuspended in EGM-2 medium and seeded on 50ul matrigel matrix (BD) in 96-well plates. Cells were analyzed starting at 4 hours posting seeding. Cells were incubated at 37 °C at 5 % CO_2.

Human DiI-Ac-LDL uptake

Human Dil-Acetylated Low Density Lipoprotein were purchased from Yeasen Company (Cat. 20606ES76, Yeasen, Shanghai, China). Cells were resuspended and grow for 24 hours and incubate with labeled LDL at concentration of 40 ug/ml for 4 hours' incubation at 37 °C. Then cells were washed by 1 % BSA PBS twice and PBS buffer for one more time and then visualized by florescence microscopy.

Gene expression profiling and statistical methods

Microarray analyses were performed by Affymetrix HG-U133 Plus-2.0 GeneChip Oligonucleotide Microarray (Affymetrix, Santa Clara, CA, USA) with standard procedure. In addition, 38 publicly available endothelial cells CEL file data were downloaded from GEO GSE43475 (http://www.ncbi.nlm.nih.gov/geo/; accession GSE) for comparison as positive controls (Aranguren et al., 2013). The following EC lines were included: human aortic ECs, human coronary artery ECs, human iliac arterial ECs, human pulmonary artery ECs, human iliac vein ECs, human pulmonary vein ECs, human hepatic artery ECs, human hepatic vein ECs, human umbilical cord vein ECs (HUVECs), and human umbilical cord artery ECs (HUAECs). For statistical analyses, Applied Biosystems Transcriptome Analysis Console 4.0 were used for quality control, data normalization, differential expression analysis and Wikipathway analysis (Slenter et al., 2018).

PLLA/PLGA polymer scaffolds

Porous polymer scaffolds consisting of 50 % polylactic-glycolic acid (PLGA) (M_n 25,000) and 50 % poly-(L-lactic acid) (PLLA) (M_n 300,000) at an average pore diameter of 250 μm were produced by Dai Gang Biology Company, which mainly followed the procedure as previously described (Levenberg et al., 2003). PLLA and PLGA porous scaffolds were widely used for PLLA could support mechanical support in three dimension while PLGA could improve cellular ingrowth by rapidly degradation. The scaffolds were cut to 4 mm*3 mm*1 mm. Before transplantation, sponges were soaked in 75 % ethyl alcohol overnight and washed three times with PBS.

Decellularized scaffolds preparation and characterization

All experiments were performed according to protocols approved by the Animal Care and Use Committee of Peking University Shenzhen Graduate School. Muscle samples for generating decellularized scaffolds were taken from the rectus femoris, biceps femoris and gluteus superficialis muscle in adult *Cavia porcellus*, which weighs 0.15 g $\pm$5 % (approximately 5 square mm in size). According to Andrea Porzionato et al.(Porzionato et al., 2015), samples were washed with ultrapure water for 24 h at 4 °C, then incubated for 1 h with 0.05 % trypsin-0.02 % EDTA at 37 °C. After washing in PBS, samples were incubated with 2 % Triton X-100-0.8 % NH_4OH for 72h at 4 °C in continuous agitation. Samples were again washed with ultrapure water for 48 h. At the end of the processing, the decellularized matrices were rinsed three times in PBS. Decellularized samples were further verified by HE staining to confirm no detectible cell structure.

Cell differentiation on scaffolds *in vitro*

Each scaffold was filled with about $1.5*10^6$ cells of which 50 % were CDH5+ cells and the rest were skeletal muscle cells.

For PLLA/PLGA polymer scaffolds, cells were suspended in 20ul of 1:1 mixture of culture medium and growth factor-reduced Matrigel (Corning). It was then injected into the PLLA/PLGA sponges followed by a 30-minute incubation at 37 °C to allow solidification of Matrigel. Culture medium (SkMCM) was then added, the sponges were detached from the bottom, and incubated at 37 °C in 5 % CO_2. The medium was changed every other day. After culturing PLLA/PLGA *in vitro* for 7 days, scaffolds were fixed in 4 % paraformaldehyde, embedded in paraffin and sectioned at 8 μm (thicker sectioning due to fragile structure).

For decellularized scaffolds, cells were suspended in 100ul of SkMCM medium and injected at multiple points scattering throughout the entire scaffolds. Three injections (each per day) were carried out to ensure that all cells were enclosed in the scaffold. Sterilized 34G needles (flat) were used. After a half hour of incubation at 37 °C in 5 % CO_2, additional SkMCM medium was added to cover the scaffolds and medium was changed every day. After culturing for 5 days, scaffolds were fixed and sectioned at 3 μm.

Implant pre-vascularized muscle graft in nude mouse

About 7-week-old male BALB/c nude mice were anesthetized by 100 mg/kg esketamine and 5 mg/kg xylazine. 3 mm*3 mm blocks in the biceps femoris muscle of nude mice were surgically removed for artificial scaffolds implantation. 30 minutes later, engineered scaffolds were implanted subcutaneously on each side of hind limb. Sutures of 8-0 prolene were used to prevent movement and mark the site of implantation. The skin was closed using surgical staples. Two weeks after implantation, the implants were retrieved. Samples were fixed in 4 % paraformaldehyde for 18 h, processed, embedded in paraffin and sectioned at 3 mm before staining.

H&E staining and immuno staining

Specimens were stained with hematoxylin-eosin staining (solarbio) following the instructions of manufacturer's kit. Immunohistochemical and immunofluorescent staining were carried out with standard protocols and adjusted with manufacturer’s instructions of specific antibodies. For immunofluorescent staining, the primary antibody was anti-hCDH5 (R&D AF938, 10 ug/ml) followed by secondary antibodies ab150129 and DAPI (Life Tech. p36966). For immunohistochemical staining, antibodies used were anti-hCDH5 antibody, anti-ETV2(Abcam ab181847, 1:50) anti-human CD31/PECAM1 (Dako), anti-GFP (Cell Signaling Technology, 2956P, 1:100), anti-Human Nuclear Antigen antibody (Abcam, ab191181, 1ug/ml) to specifically mark human cells, HRP conjugate Secondary Antibody A16005, A24537 and A16072 (Life Tech., usually at 1:2000). For mouse anti-human PECAM1 antibody, anti-mouse IgG (H+L) secondary antibody were specifically used for blocking. DAB Substrate Kit ab64238 was used for color reaction.

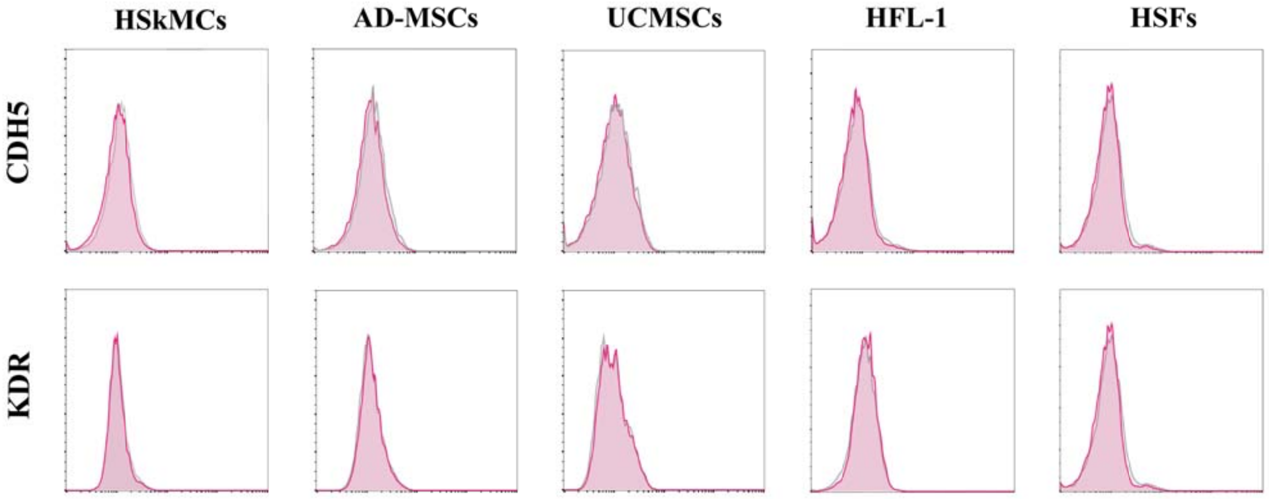

Figure S1. Comparison of endothelial potential before ETV2 transduction. There was no detectable expression of CDH5/ CD144 (A) or KDR (B) in HSkMCs, ADMSCs, UCMSCs, HFL-1, HSFs without ETV2-virus infection.

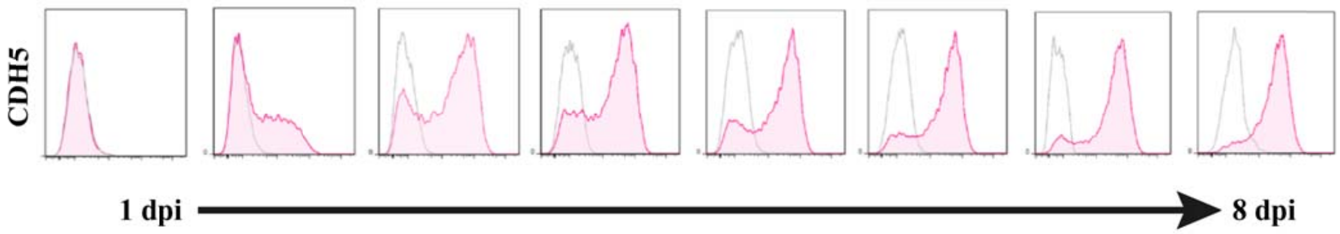

Figure S2. Temporal expression profile of endothelial maker CDH5 in ETV2 infected adipose derived mesenchymal stem cells. Compared to ETV2-HSkMCs, induced expression of CDH5 in adipose derived mesenchymal stem cells was less efficient.

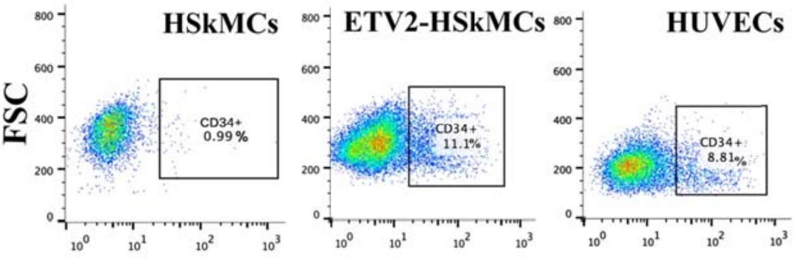

Figure S3. CD34 positive cells were detected from ETV2-HSkMCs with a similar ratio of HUVECs.

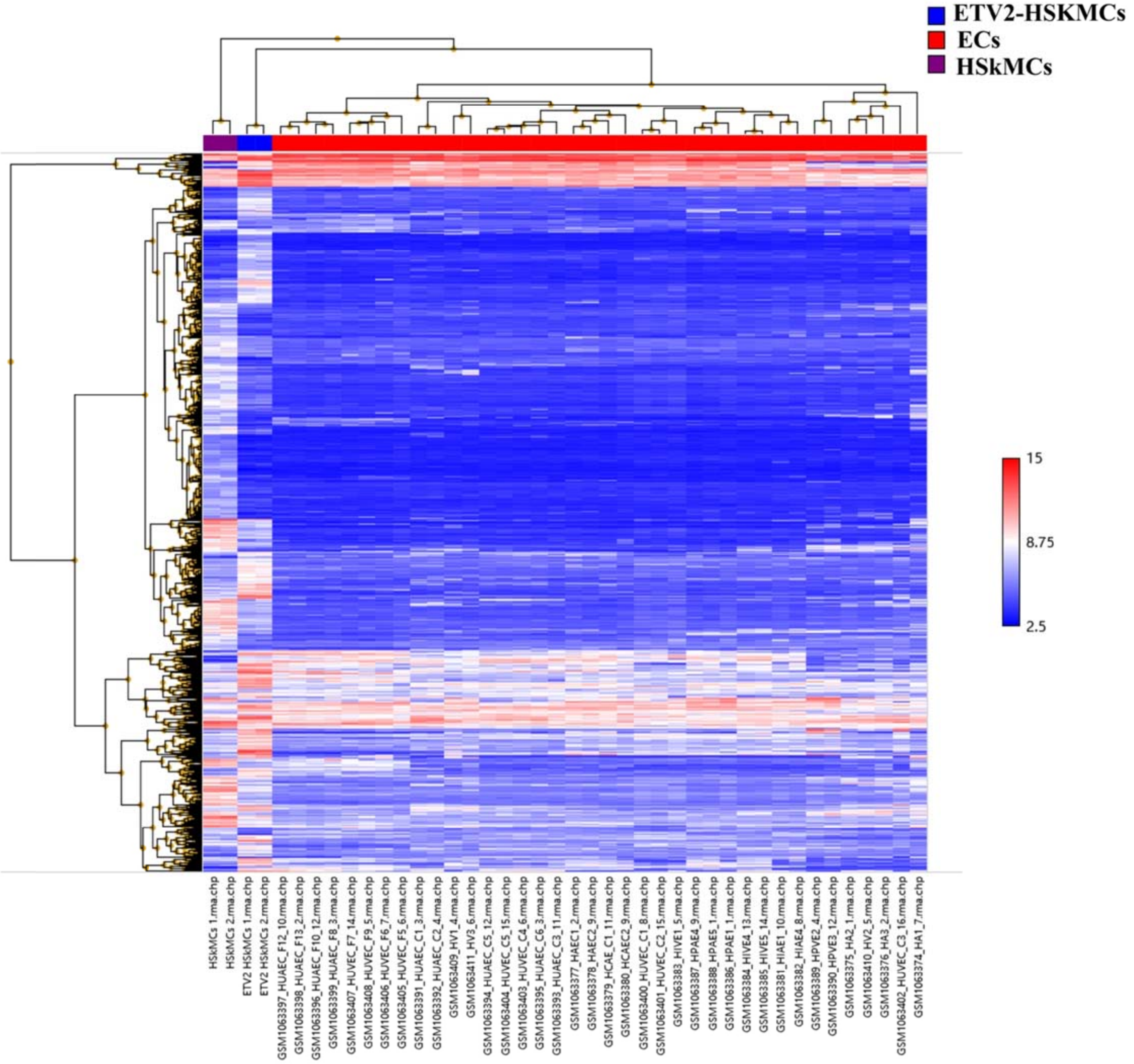

Figure S4. Clustering analysis of microarray data. Transcripts with over 4-fold expressional changes between HSkMCs and ETV2-SKMCs were used for comparing. The results showed that the distance between ETV2-SKMCs and endothelial cells is shorter than that between HSkMCs and endothelial cells.

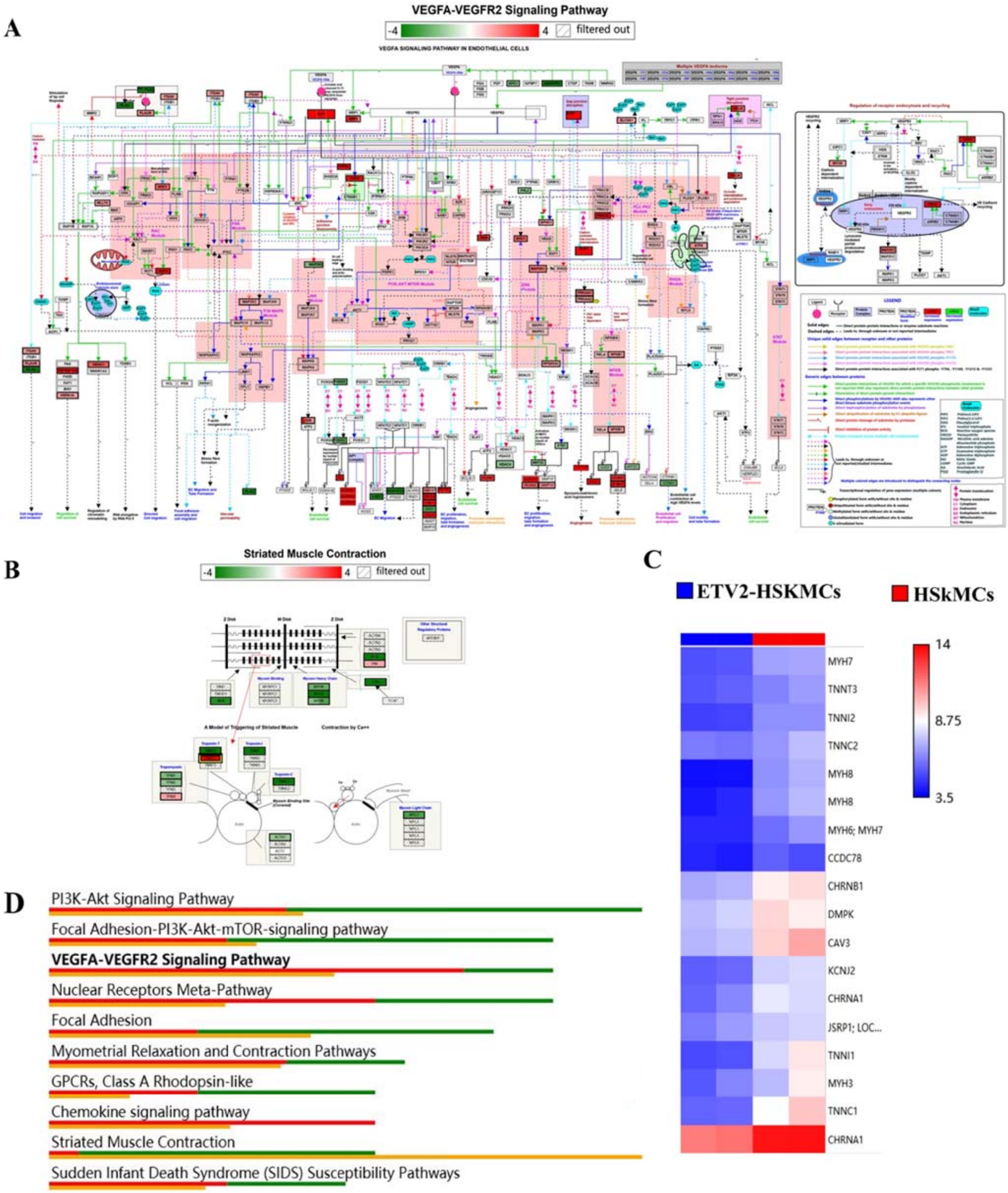

Figure S5. Microarray and pathway analysis. Wikipathway analysis of VEGF-VEGFR2 pathway (A) and striated muscle contraction pathway (B). It was found that 37 out of 50 (75 %) of differentially expressed genes in the

VEGF-VEGFR2 pathway were upregulated, while 13 out of 16 (81 %) of differentially expressed genes in the striated muscle contraction pathway were downregulated. (C) Some muscle functional genes were down regulated after ETV2 expression. (D) Top ranked pathways revealed by the most differentially expressed genes with over 4-fold changes between HSkMCs and ETV2- HSkMCs, suggesting possible mechanisms of ETV2 induction. Up-regulated by ETV2 expression is labeled by red, down-regulated by green, and yellow bar stands for significance.

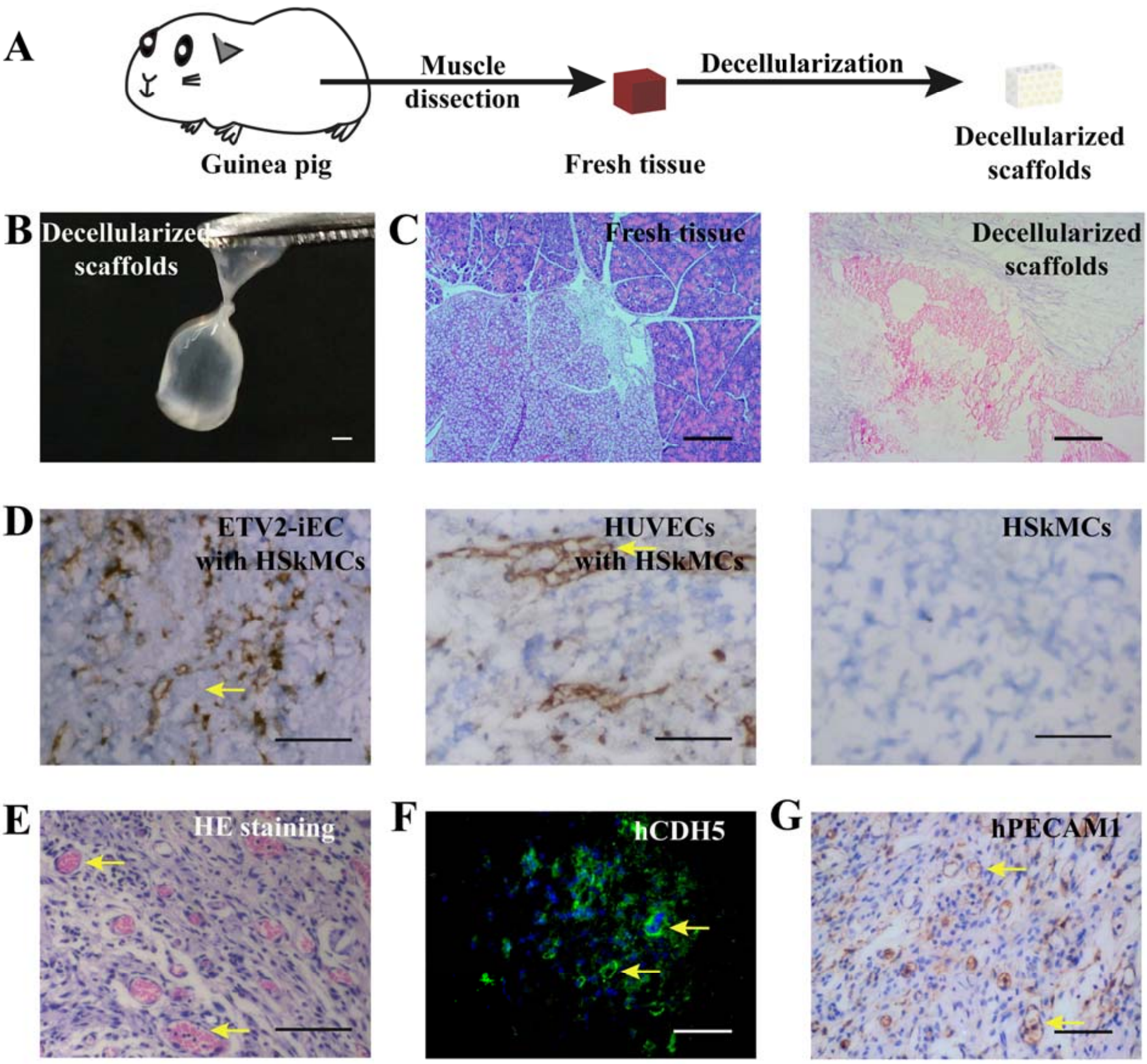

Figure S6. Analysis of engineered muscle on decellularized scaffolds. (A) Scheme of producing decellularized scaffolds from Guinea pig. (B) Decellularized scaffolds were transparent after enzymatic digestion and detergent perfusion, scale bar: 1mm. (C) H&E staining of Guinea pig muscle without decellularized process and with process. (D) Analysis of engineered muscle tissue with ETV2-iEC on decellularized scaffolds *in vitro*. (E) H&E staining of decellularized scaffolds engineered tissues after 15 days' implantation. Immunostaining against human CDH5(F) and human PECAM1 (G). Yellow arrows indicate vessel structures. Scale bars in C, D, E, F and G: 100 µm.

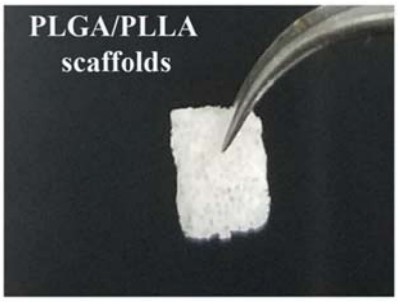

Figure S7. Synthetic scaffold composed of PLLA and PLGA was porous material, 4 mm*3 mm.

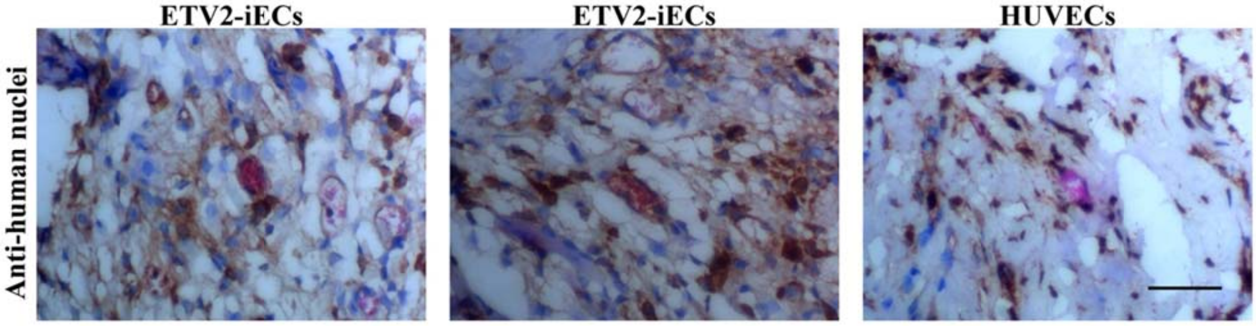

Figure S8. Anti-human nuclei staining. DAB staining (brown) of anti-human nuclei revealed human origin of the implanted cells 2 weeks after transplantation. Hematoxylin (blue) stained both human and mouse nuclei. A brief (one second) staining by eosin dyeing (pink) showed blood cells without interfering DAB signals. Scale bar: 100 µm.

**GAPDH**	Forward	AGCCTCAAGATCATCAGCAA
	Reverse	CCATCACGCCACAGTTTCC
**ACTB**	Forward	CACCATTGGCAATGAGCGGTTC
	Reverse	AGGTCTTTGCGGATGTCCACGT
**ETV2**	Forward	GAAGGAGCCAAATTAGGCTTCT
	Reverse	GAGCTTGTACCTTTCCAGCAT
**CDH5**	Forward	CTCTGCATCCTCACCATCAC
	Reverse	GAGTTGAGCACCGACACATC
**KDR**	Forward	CGGCTCTTTCGCTTACTGTT
	Reverse	TCCTGTATGGAGGAGGAGGA
**TAL1**	Forward	TTCCCTATGTTCACCACCAA
	Reverse	AAGATACGCCGCACAACTTT
**PECAM1**	Forward	ACTGCACAGCCTTCAACAGA
	Reverse	TTTCTTCCATGGGGCAAG
**TEK**	Forward	GCTTGCTCCTTTCTGGAACTGT
	Reverse	CGCCACCCAGAGGCAAT

Supplementary table 1. Gene-specific primers used in real-time PCR.

Aranguren, X.L., Agirre, X., Beerens, M., Coppiello, G., Uriz, M., Vandersmissen, I., Benkheil, M., Panadero, J., Aguado, N., Pascual-Montano, A., *et al.* (2013). Unraveling a novel transcription factor code determining the human arterial-specific endothelial cell signature. Blood *122*, 3982-3992.
Levenberg, S., Huang, N.F., Lavik, E., Rogers, A.B., Itskovitz-Eldor, J., and Langer, R. (2003). Differentiation of human embryonic stem cells on three-dimensional polymer scaffolds. Proc Natl Acad Sci U S A *100*, 12741-12746.
Lindgren, A.G., Veldman, M.B., and Lin, S. (2015). ETV2 expression increases the efficiency of primitive endothelial cell derivation from human embryonic stem cells. Cell Regen (Lond) *4*, 1.
Porzionato, A., Sfriso, M.M., Pontini, A., Macchi, V., Petrelli, L., Pavan, P.G., Natali, A.N., Bassetto, F., Vindigni, V., and De Caro, R. (2015). Decellularized Human Skeletal Muscle as Biologic Scaffold for Reconstructive Surgery. Int J Mol Sci *16*, 14808-14831.
Slenter, D.N., Kutmon, M., Hanspers, K., Riutta, A., Windsor, J., Nunes, N., Melius, J., Cirillo, E., Coort, S.L., Digles, D., *et al.* (2018). WikiPathways: a multifaceted

pathway database bridging metabolomics to other omics research. Nucleic Acids Res *46*, D661-D667.
